# Bayesian analysis of depth resolved OCT attenuation coefficients

**DOI:** 10.1038/s41598-021-81713-7

**Published:** 2021-01-26

**Authors:** Lionel D. Fiske, Maurice C. G. Aalders, Mitra Almasian, Ton G. van Leeuwen, Aggelos K. Katsaggelos, Oliver Cossairt, Dirk J. Faber

**Affiliations:** 1 Engineering Science and Applied Mathematics, Northwestern University, Chicago, IL USA; 2 Computer Science, Computational Photography Lab, Northwestern University, Chicago, IL USA; 3 Department of Biomedical Engineering and Physics, Academic Medical Center, University of Amsterdam, Amsterdam, The Netherlands; 4 Electrical and Computer Engineering, Image and Video Processing Lab, Northwestern University, Chicago, IL USA

**Keywords:** Biological physics, Biomedical engineering

## Abstract

Optical coherence tomography (OCT) is an optical technique which allows for volumetric visualization of the internal structures of translucent materials. Additional information can be gained by measuring the rate of signal attenuation in depth. Techniques have been developed to estimate the rate of attenuation on a voxel by voxel basis. This depth resolved attenuation analysis gives insight into tissue structure and organization in a spatially resolved way. However, the presence of speckle in the OCT measurement causes the attenuation coefficient image to contain unrealistic fluctuations and makes the reliability of these images at the voxel level poor. While the distribution of speckle in OCT images has appeared in literature, the resulting voxelwise corruption of the attenuation analysis has not. In this work, the estimated depth resolved attenuation coefficient from OCT data with speckle is shown to be approximately exponentially distributed. After this, a prior distribution for the depth resolved attenuation coefficient is derived for a simple system using statistical mechanics. Finally, given a set of depth resolved estimates which were made from OCT data in the presence of speckle, a posterior probability distribution for the true voxelwise attenuation coefficient is derived and a Bayesian voxelwise estimator for the coefficient is given. These results are demonstrated in simulation and validated experimentally.

## Introduction

Optical coherence tomography (OCT) is an imaging modality which allows for the visualization of internal structures of tissues and other translucent materials volumetrically. OCT images give a practitioner insight into qualitative structural information such as layer structure and morphology. However, the extraction of reliable quantitative information from these tissue volumes is an area of current research. One quantitative measure of interest is the rate of signal decay in depth known as the attenuation coefficient^1^. The attenuation coefficient compounds effects of absorption and scattering losses in depth which can be related to physiological properties such as blood content and tissue organization^1–3^. Currently, methods to extract the attenuation coefficient fall into one of two categories: layerwise extraction through curve fitting^4^ and depth resolved or voxelwise extraction^5^.

In the layerwise approach, the layers of media are segmented, and then an exponentially decaying model is fit to each A-scan of the OCT signal in the least squares sense^3–5^. From this perspective, the attenuation coefficient is a bulk measure which assigns a single, deterministic number to each segment of an A-scan. However, a measured A-scan will contain fluctuations due to speckle^6,7^. OCT speckle is the voxel-to-voxel variation of OCT amplitude, due to random variations in the spatial position of scattering particles within the imaging voxel. Randomly placed scatterers within the voxels will thus return scattered fields with random amplitude and phase—leading to intensity fluctuations at the detector. While the origin of speckle is deterministic at the microscopic level, in practice the measured signal is well modeled as a realization of a random process equivalent to randomly varying the exact microscopic position of the scattering particles in the bulk of the media^8^. One common technique to overcome the speckle variations is lateral averaging^2,3,9^, where neighboring A-scans are averaged together prior to fitting. Lateral averaging can be an effective technique at reducing speckle variations but at a severe cost to lateral resolution. If the sample is not perfectly static, as is the case in liquid samples with particles undergoing Brownian motion or sufficiently dynamic living samples, consecutive A-scans taken at the same location can be averaged together to reduce speckle variations at the cost of effective acquisition time^10^. In either case, the layerwise fitting assumes complete uniformity in the composition and statistics of the layer segment in depth and lateral averaging makes the same assumption over a volumetric region.

A depth resolved (DR) approach, initially developed for ultrasound image quantification^11^, was adapted by Vermeer for use in OCT and has become popular in recent years^12–14^. This approach removes the assumption of material uniformity in depth and allows variations in the attenuation coefficient in three dimensions. The DR approach assumes the material is weakly absorbing, making this technique related to voxelwise OCT scattering parameter inference methods^15–17^ which have a long history in OCT signal processing. This method has been further refined by Liu^18^ to better handle boundary effects caused by finite imaging depth. In either formulation, reconstructions of the attenuation coefficient will be highly variable due to the influence of speckle^14^. Thus, as before, lateral averaging is often still employed to get a more consistent result^12^. Conceptually, the DR approach allows one to recover some amount of the natural variability of optical properties within the tissues. While the advantages of the DR approach are manifest, the result of this approach in the presence of intensity variations due to speckle leads to reconstructions in which the recovered attenuation coefficient itself has large variations.

The propagation of speckle variation into the recovery of an otherwise deterministic coefficient has clear implications for the accuracy of the attenuation parameter inferred at a single voxel. Since the exact measured intensity is effectively random, one can in general expect the inferred coefficient to be effectively random as well. One way to handle the inference of parameters in these circumstances would be to adopt a Bayesian perspective. In this paradigm, instead of simply seeking an estimate for the value, one seeks the posterior distribution, which quantifies how probable each attenuation value is^19^. In these methods, accurate physical models about measurement uncertainty are combined with prior information about the objects which are being measured. Utilizing the posterior distribution allows for the identification of estimation biases and the quantification of uncertainty by giving access to statistics about the inferred attenuation coefficient. A better understanding of uncertainty can have direct clinical implications by helping to inform practitioners of how much they can trust a given inference. Furthermore, this approach opens the door to probabilistic tissue classification tasks such as tumor grading where the likelihood of various outcomes must be compared.

In this manuscript we model the effect of speckle on the inference of OCT attenuation coefficients using a Bayesian approach. The interaction between the DR reconstruction technique and the speckle variation is considered, and a probability distribution for the measurements made under physically realistic speckle variations is derived. Following this, we derive a prior distribution for a simple system using statistical mechanic principles. Finally, we combine these to derive a probability distribution for the attenuation coefficient itself and define a Bayesian voxelwise estimator for the mean attenuation coefficient. These results are then demonstrated in simulation and experimentally.

### Paper structure

The goal of this work is to construct the *posterior distribution* for the voxelwise attenuation coefficient and to validate it using numerical experiments and tissue phantom measurements. The posterior distribution assigns a meaningful probability to every possible value of the true attenuation coefficient. Here, the true attenuation coefficient is defined as the attenuation coefficient of the mean OCT signal without speckle fluctuations. Using the existing DR method^12^, the attenuation coefficient at each voxel can be estimated from the measured OCT signal. These depth resolved estimates are denoted as $\hat{\mu }$. These estimates depend on the intensity at each voxel which fluctuates due to speckle. Because of these voxelwise fluctuations, the estimated value of the attenuation coefficient at that point will likely differ from the true coefficient. The posterior probability distribution gives the probability that the true value of the attenuation coefficient is equal to $\mu _{oct}$ given that our depth resolved estimate was equal to $\hat{\mu }$.

Mathematically, the posterior distribution can be written as the conditional probability distribution $P\left( \mu _{oct} \big | \hat{\mu }\right)$. Conditional probabilities can be rewritten as product of two easier to derive probability distributions using Bayes’ theorem. This theorem states that the posterior distribution is given by,

1 $$\begin{aligned}&P\left( {\mu }_{oct} \big | \hat{\mu }\right) = \frac{P\left( \hat{\mu }\big | {\mu }_{oct} \right) P\left( {\mu }_{oct} \right) }{P\left( \hat{\mu }\right) }. \end{aligned}$$

In this expression, $P\left( \hat{\mu }\big | {\mu }_{oct} \right)$ is called the likelihood function which represents the probability of estimating $\hat{\mu }$ given that the true attenuation coefficient is equal to ${\mu }_{oct}$. The distribution denoted by $P\left( {\mu }_{oct} \right)$ is called the prior distribution for the unknown $\mu _{oct}$. The prior probability allows the incorporation of additional information into the statistical model and is often used as a way to establish bounds or to bias solutions towards realistic values. The marginal probability $P\left( \hat{\mu }\right)$ is a normalizing factor and can be computed via integration. Using this relation, we can find the posterior distribution by solving two easier problems: finding the likelihood function and finding the prior distribution.

Before these two distributions can be derived we must first have a mathematical model for the measured OCT signal so we can make depth resolved attenuation coefficient estimates. In “Modeling intensity decay”, a model which describes the mean signal decay is given. This model assumes that the measurements are made on a weakly absorbing medium and that the majority of measured light is single scattered. Next, in “A statistical model of the OCT amplitude and intensity”, the effect of speckle on this OCT signal is considered and the probability distribution for the measurement is given. The likelihood function, $P\left( \hat{\mu }\big |\mu _{oct} \right)$, is derived in “Analyzing the DR reconstruction distribution” by analyzing the speckle variations and is verified experimentally in “Experimental verification and results” by measuring the distribution of depth resolved attenuation coefficient estimates for a very homogeneous phantom. In “Constructing a prior distribution”, the prior probability, $P(\mu _{oct})$, is derived using basic physical principles. This prior gives the background probability for finding a particular value of $\mu _{oct}$ at any point in the sample without any additional measurement information. Following this we define a Bayesian estimator for the attenuation coefficient in “Bayesian parameter estimator”. In “Simulation results” we simulate OCT signals with realistic variations to test our assumptions and statistical model.

## Methods

### Modeling intensity decay

In many practical OCT systems, the decay of the OCT intensity with depth can be adequately described using a single exponential decay model^5,20,21^ , understanding the form of the OCT signal is necessary prior to understanding the attenuation itself. The attenuation coefficient is a material property, which depends on the absorption and scattering properties of tissue and is not a function of the measurement system. However, several system dependent factors can also contribute to measured signal attenuation such as the confocal point spread function and the sensitivity roll off function for OCT systems based on detection in the Fourier domain^4,22^. A model which takes all of these effects into account was described in detail in earlier work^20,22^. Typically for an OCT system, the signal decay due to the confocal PSF and the sensitivity roll off function can be independently measured, and subsequently, the resulting OCT data can be corrected for these effects. For the sake of analysis, we will assume that the measured signal has already been calibrated for these system dependent effects. A more thorough discussion of this can be found in Supplemental Information S3.

We denote the corrected OCT signal at depth *z* as $I(z ; \mu _{b,NA}(z), \mu _{oct}(z))$ where $\mu _{b,NA}(z)$ is the depth dependent back-scattering coefficient (the probability per unit length that light is back-scattered into the detection numerical aperture). The depth dependent attenuation coefficient, $\mu _{oct}(z)$, and the back-scattering coefficients depend on both the scattering coefficient $\mu _s$ and the absorption coefficient $\mu _a$. These coefficients describe the probabilities of scattering and absorption per unit length, respectively. For weakly scattering samples, with negligible contributions from multiple scattered light, $\mu _{oct}=\mu _s+\mu _a$.

Following Vermeer^12^, we further assume that the tissue is very weakly absorbing ($\mu _a \approx 0$), and, a constant fraction of the attenuated light is back-scattered at every point in the tissue. We denote this fraction as $\beta _{NA}$ and define $\mu _{b,NA} = \beta _{NA}\mu _{oct}$. Physically, this implies that the system is highly scattering dominant, i.e., there is very little absorbed light in the system when compared to the total attenuated light. Lastly, we assume the measurements are made with a fixed axial resolution denoted by $\Delta z$. Combining these assumptions the discretized quantity

2 $$\begin{aligned} I_{N} =I(N\Delta z ; \mu _{oct}(N)) = I_{inc} \beta _{NA} \mu _{oct} (N) \exp \left( -2 \sum _{i=1}^N \mu _{oct}(i\Delta z) \Delta z \right) \end{aligned}$$

is defined which describes the mean value of the OCT signal with depth in a certain region at depth $z = N \Delta z$ where N is the pixel index and given an incident intensity $I_{inc}$ . We use the shorthand $\mu _{oct} (N) = \mu _{oct} (N\Delta z)$. Provided that the inverse of the attenuation coefficient is relatively small compared with the pixel size, its value is given by^12^

3 $$\begin{aligned} \mu _{oct} (N) = \frac{I_N}{\sum _{i=N+1}^\infty 2\Delta z I_i }. \end{aligned}$$

As recently noted by Liu^18^, the tail of the series in the denominator in Eq. (3), meaning all of the terms in the sum after some large term *K*, can be computed when an estimate for an attenuation coefficient at that point in the sample is available. This is given by

4 $$\begin{aligned} {\sum _{i=K+1}^\infty 2 \Delta z I_i } = \frac{I_K}{\mu _{oct}(K)}. \end{aligned}$$

### A statistical model of the OCT amplitude and intensity

The measured OCT signal is the amplitude of the backscattered field, which contains contributions from scatterers within the measurement volume, each contributing to the resulting field with their respective random amplitude and phase. This scattering results in OCT signal amplitude fluctuations called speckle. When there are sufficiently many scattering events within a single voxel, the speckle is called fully developed^23^ and the measured signal becomes effectively random. In this case, the statistics for the signal amplitude, *A*, are well described by the Rayleigh distribution^8^ given by

5 $$\begin{aligned} P_{amp}(A|\langle I \rangle ) = \frac{A}{\langle I \rangle }\exp \left( - \frac{A^2}{\langle I \rangle } \right) \end{aligned}$$

where $\langle I \rangle$ denotes the mean intensity value. This formula gives the probability of measuring amplitude *A* when the mean signal is given by $\sqrt{\langle I \rangle }$. When OCT measurements are made, typically intensity is measured and not amplitude. Given a Rayleigh distributed amplitude of the form given in Eq. (5) it can be shown that the intensity^24^, which is the square of the amplitude, follows

6 $$\begin{aligned} P_{int}(I|\langle I \rangle ) =&\frac{1}{\langle I \rangle }\exp \left( - \frac{I}{\langle I \rangle } \right) \end{aligned}$$

which is an exponential distribution with parameter $\langle I \rangle$.

### Analyzing the DR reconstruction distribution

This section considers the estimation of $\mu _{oct}(N)$ from intensity measurements in the presence of speckle modeled by Eq. (6). In this case, instead of measuring the mean intensity $\langle I_N \rangle$ directly we can only measure $I_N$ which is exponentially distributed with parameter $\langle I_N \rangle$. Because the constituent parts of Eq. (3) are now random, the estimate will be itself a random variable. The estimated random variable is denoted as

7 $$\begin{aligned} \hat{\mu }(N) = \frac{I_N}{\sum _{i=N+1}^\infty 2 \Delta z I_i} \end{aligned}$$

Following Vermeer^12^ we consider the attenuation coefficient at the $N^{th}$ point and truncate the series in the denominator at *M* which in practice corresponds to the maximum imaging depth $Z_{max}$ with $M>N$ giving

8 $$\begin{aligned} \hat{\mu }(N) \approx \frac{I_N}{\sum _{i=N+1}^M 2 \Delta z I_i}. \end{aligned}$$

Consider the denominator, and let,

9 $$\begin{aligned} D_N = \sum _{i=N+1}^M 2 \Delta z I_i. \end{aligned}$$

The variable $D_N$ is the sum of $M - (N+1)$ independent exponentially distributed random variables $I_i$, taken from distributions parameterized only with average $\langle I_i \rangle$. Thus, $D_N$ will be distributed as a hypoexponential distribution and has mean

10 $$\begin{aligned} \langle D_N \rangle = \sum _{i=N+1}^M 2 \Delta z \langle I_i \rangle , \end{aligned}$$

because the $I_i$’s are independent. If *M* is sufficiently larger than *N*, Eq. (4) implies that

11 $$\begin{aligned} \langle D_N \rangle \approx \frac{\langle I_N \rangle }{\mu _{oct}(N)} . \end{aligned}$$

It is known that reconstruction artifacts^12,18^ make the inferred coefficient unreliable near the deepest point of an A-scan. In practice, the reconstructed attenuation coefficient made from this approach must be discarded near the bottom of a scan and estimated using a different method^18^.

One useful measure of how much a random variable deviates from the mean called the coefficient of variation, and is denoted $C_v$. This quantity is defined as the standard deviation divided by the mean. It can be shown that for a hypoexponential variable the coefficient of variation is always less than 1 as shown in the Supplemental Information S1. In practice, we find that $C_{v} \ll 1$ as demonstrated in Fig. 4 and described in detail in “Results”.

Next, letting

12 $$\begin{aligned} \eta _N := D_N - \langle D_N \rangle \end{aligned}$$

allows formula (8) to be rewritten as

13 $$\begin{aligned} \hat{\mu }(N) = \frac{I_N}{ \langle D_N \rangle } \left( \frac{1}{1 + \frac{\eta _N}{\langle D_N \rangle } } \right) . \end{aligned}$$

Because $\eta$ has zero mean with a very small $C_v$ one can expect $\frac{\eta _N}{\langle D_N \rangle }$ to be small. Using this as justification, consider the Taylor approximation

14 $$\begin{aligned} \hat{\mu }(N) \sim \frac{I_N }{\langle D_N \rangle } \left( 1 - \frac{\eta _N}{\langle D_N \rangle } \right) + \mathcal O \left( \frac{\eta _N^2}{ \langle D_N \rangle } \right) . \end{aligned}$$

At leading order, the reconstruction of the attenuation coefficient is given by

15 $$\begin{aligned} \hat{\mu }(N) \approx \frac{I_N}{\langle D_N \rangle }. \end{aligned}$$

Intuitively, this means the denominator of Eq. (8) is approximately constant at the scale set by the mean. Therefore, the probability distribution of $\hat{\mu }$ will be given by rescaling the distribution of $I_N$. Rescaling Eq. (6) yields

16 $$\begin{aligned} P\left( \hat{\mu }(N) \big | \langle I_N\rangle , \langle D_N\rangle \right) = \frac{\langle D_N \rangle }{\langle I_N \rangle } \exp \left( - \langle D_N \rangle \frac{\hat{\mu }(N)}{\langle I_N \rangle } \right) . \end{aligned}$$

Next, using the approximation for the tail of *D* given in Eq. (11) with $K=N$ and substituting $\frac{\langle I_N \rangle }{\langle D_N \rangle }$ with $\mu _{oct}(N)$ yields the probability distribution

17 $$\begin{aligned} P\left( \hat{\mu }(N) \big | \mu _{oct}(N) \right) \approx \frac{1}{\mu _{oct}(N)} \exp \left( - \frac{\hat{\mu }(N)}{\mu _{oct}(N)} \right) . \end{aligned}$$

Therefore, the reconstructed coefficient at leading order will be exponentially distributed around the mean attenuation parameter. The accuracy of this estimate is demonstrated in Fig. 4.

This approach can be extended to the time-averaged case, where *k* independent co-registered measurements have been made. To do this, first the *k* estimates for the attenuation coefficient, denoted by $\hat{\mu }_i(N), \ \ i=1,2m,\ldots ,k$, should be constructed using Eq. (8). Then, assuming the measurements are independent, the likelihood is given by

18 $$\begin{aligned} P\left( \hat{\mu }_1\left( N \right) , \ldots , \hat{\mu }_k\left( N \right) \big | \mu _{oct}(N) \right) = \prod _{i=1}^k P\left( \hat{\mu }_i\left( N \right) \big | \mu _{oct}(N) \right) = \left( \frac{1}{\mu _{oct}(N)}\right) ^k\exp \left( - \frac{\sum _{i=1}^k \hat{\mu }_i(N)}{ \ \mu _{oct }(N)} \right) . \end{aligned}$$

### Constructing a prior distribution

In this section, a prior distribution for the variation in attenuation coefficient in a layer is derived based on physical principles. As an initial theoretical step we consider a simplified media of dispersed scattering particles with negligible absorption. Following Chandrasekhar^25^ it is assumed that the system is a single layer, with $N_p$ dispersed particles throughout. Let

19 $$\begin{aligned} \gamma = \frac{[\text {voxel}]_{vol}}{[\text {scan}]_{vol}} \end{aligned}$$

be the ratio of the volume of a single voxel to the volume of the entire scanned layer. Provided that $[\text {voxel}]_{vol} \ll [\text {scan}]_{vol}$ the probability of finding n particles inside the volume defined by a single voxel is given by the Poisson distribution

20 $$\begin{aligned} P_{vox}(n):=P(\text {n particles inside voxel}) \sim \frac{ \langle n \rangle ^ n e^{- \langle n \rangle }}{n!}, \end{aligned}$$

where the mean particle number $\langle n \rangle = N_p \gamma$. For very large particle counts, $N_p \rightarrow \infty$, the Poisson distribution is well approximated as

21 $$\begin{aligned} P_{vox}(n) = \frac{\exp \left( -\frac{\left( n - \langle n \rangle \right) ^2}{2 \langle n \rangle } \right) }{\sqrt{2\pi \langle n \rangle }}. \end{aligned}$$

Since absorption can be ignored, the attenuation coefficient can then be computed as $\mu \sim \sigma _{scat} \frac{n}{[voxel]_{vol}}$ and the mean coefficient as $\langle \mu \rangle =\sigma _{scat} \frac{ \langle n \rangle }{[voxel]_{vol}}$, where $\sigma _{scat}$ is the scattering cross section of a particle. Therefore, $P(\mu )$ is a rescaled version of the probability distribution in Eq. (21) given by

22 $$\begin{aligned} P(\mu ) = \frac{1}{\sqrt{2 \pi \zeta \langle \mu \rangle }} \exp \left( -\frac{\left( \mu - \langle \mu \rangle \right) ^2}{2 \zeta \langle \mu \rangle } \right) \end{aligned}$$

where $$\zeta = \frac{\sigma _{scat}}{[voxel]_{vol}}$$.

### Deriving the posterior for the reconstructed attenuation coefficients

The posterior distribution for the attenuation coefficient at depth *N* can be now derived by plugging in the Eqs. 18 and 22 into Eq. (1). Thus, our posterior distribution is proportional to

23 $$\begin{aligned} P\left( \mu _{oct}(N)\big | \hat{\mu }_1(N) \cdots \mu _k(N) \right) \propto \left( \frac{1}{\mu _{oct}(N)}\right) ^k\exp \left( - \frac{\sum _{i=1}^k \hat{\mu }_i(N)}{ \ \mu _{oct}(N)} \right) \frac{ 1}{\sqrt{2 \pi \zeta \langle \mu _{oct} \rangle }} \exp \left( -\frac{\left( \mu _{oct}(N)- \langle \mu _{oct} \rangle \right) ^2}{2 \zeta \langle \mu _{oct} \rangle } \right) \end{aligned}$$

where *k* is the number of co-registered scans and $\langle \mu _{oct} \rangle$ is the layer mean of the DR estimates. The specific proportionality constant is given by integrating the numerator of Eq. (1) over all possible values of $\mu _{oct}$. Considering the case where only a single independent scan can be made the posterior distribution for the attenuation coefficient at depth *N* is given by

24 $$\begin{aligned} P\left( \mu _{oct}(N)\big | \hat{\mu }(N) \right) \propto \frac{1}{\mu _{oct}(N)}\exp \left( - \frac{\hat{\mu }(N)}{ \ \mu _{oct}(N)} \right) \frac{ 1}{\sqrt{2 \pi \zeta \langle \mu _{oct} \rangle }} \exp \left( -\frac{\left( \mu _{oct}(N)- \langle \mu _{oct} \rangle \right) ^2}{2 \zeta \langle \mu _{oct} \rangle } \right) \end{aligned}$$

**Figure 1:**
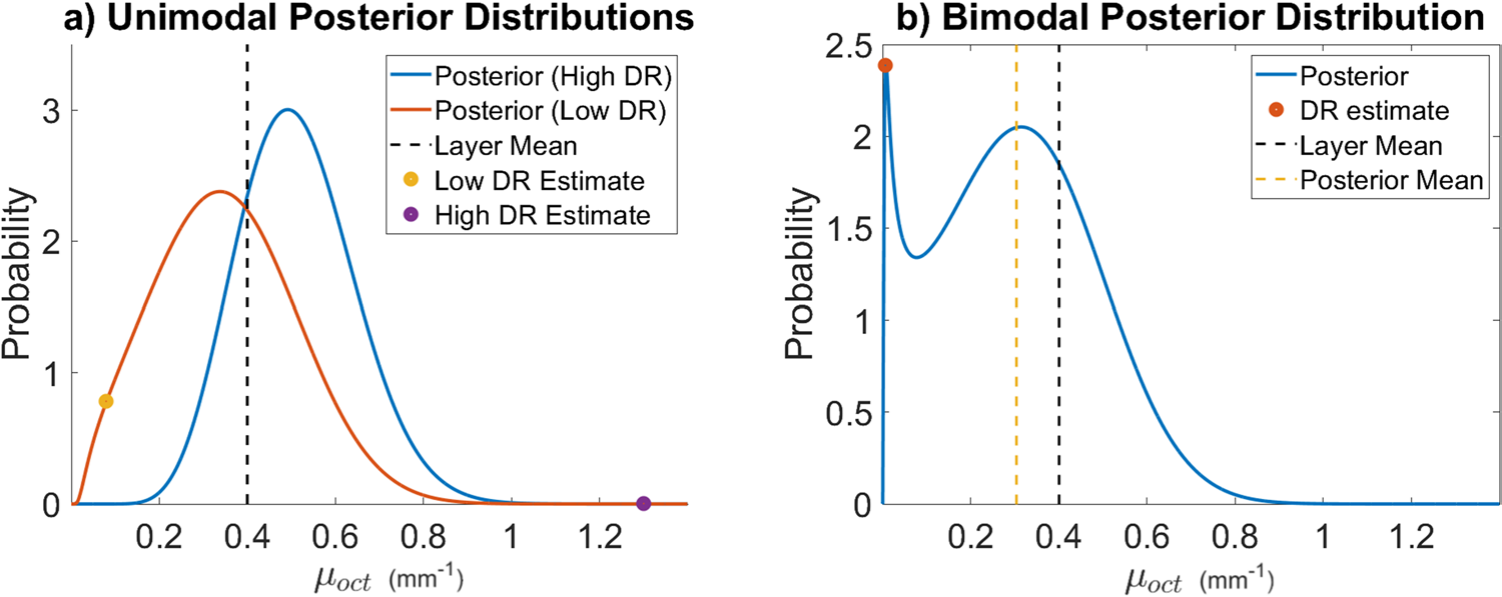
This figure shows realizations of the posterior distribution for the attenuation coefficient given in Eq. (24) for different values of $\hat{\mu }$. Both the simulations and figure creation were done in Matlab 2019a^27^, https://www.mathworks.com/. (**a**) This panel shows two unimodal reconstructed posterior distributions. With these distributions, the true parameter is much more likely than the DR estimate. This posterior was constructed with a layer mean of $\langle \mu _{oct} \rangle = 0.4{\text { mm}}^{-1}$, $\zeta = 6.87\times10^{-2}{\text { mm}}^{-1}$ and a DR estimates of $\hat{\mu }= 0.08{\text { mm}}^{-1}$ and $\hat{\mu }= 1.3 {\text { mm}}^{-1}$. (**b**) This panel shows a constructed posterior distribution which is Bi-modal and has two local maxima. For a given layer mean, the constructed distribution develops a second peak if the DR estimate used to construct the posterior is sufficiently small. This second peak can make the Maximum a Posteriori difficult due to non-convexity. In many cases, the maximum value of the Posterior distribution may sit very near the origin on this second peak. As demonstrated in this panel, often the total amount of probability mass under the addition peak is relatively small, meaning that while the initial peak is overwhelmingly the maximum likelihood. Thus, the Maximum of the posterior distribution is a poor representation for the distribution itself. In these cases an estimate for the mean is a better choice. This posterior was constructed with a layer mean of $\langle \mu _{oct} \rangle = 0.4 {\text { mm}}^{-1}$, $\zeta = 6.87\times10^{-2} {\text { mm}}^{-1}$, and a DR estimate of $\hat{\mu }= 0.015 {\text { mm}}^{-1}$.

This distribution describes the probability of the mean coefficient at voxel *N*. Assuming that each voxel is independent, a joint posterior distribution for the attenuation coefficient map for the entire A, B or C scan can be written as

25 $$\begin{aligned} P\left( \pmb \mu _{oct} \big | \pmb {\hat{\mu }} \right) \propto \prod _{i=1}^R P\left( \mu _{oct}(i)\big | \hat{\mu }(i) \right) \end{aligned}$$

where R is the total number of voxels in the scan, $\pmb \mu _{oct}$ is an $R \times 1$ vector of true coefficients and $\pmb {\hat{\mu }}$ is the $R \times 1$ vector of voxelwise estimates for the attenuation coefficient. Figure 1 shows two posterior distributions plotted using Eq. (24) which use two different values for the DR estimate. These examples demonstrate the impact that the initial DR estimate has on the shape and position of the posterior distribution for the attenuation coefficient.

### Bayesian parameter estimator

In Bayesian formulations of parameter estimation problems, when a single number prediction for the coefficient must be made, a Maximum a Posteriori (MaP) approach is often employed^14,26^. This approach gives the attenuation coefficient which maximizes the posterior distribution. However, as can be seen in Fig. 1 for sufficiently small DR estimates, the posterior distribution becomes bimodal and the MaP estimate will nearly coincide with the low DR estimate for the attenuation coefficient. As demonstrated in Fig. 1b this peak is relatively narrow and contains little probability mass. Because of this, the maximum a posteriori is a bad representation of the entire probability distribution. The mean of the posterior distribution is agnostic to the bimodality of the distribution and provides a more stable and realistic estimate for the attenuation parameter. Therefore, when a single value estimate is desired, the quantity

26 $$\begin{aligned} \hat{\mu }_{mean} (N) := \int _{\mathbb R^+} \mu _{oct} P\left( \mu _{oct}(N)\big | \hat{\mu }(N) \right) d\mu _{oct}. \end{aligned}$$

can be computed.

## Results

### Experimental verification and results

**Figure 2:**
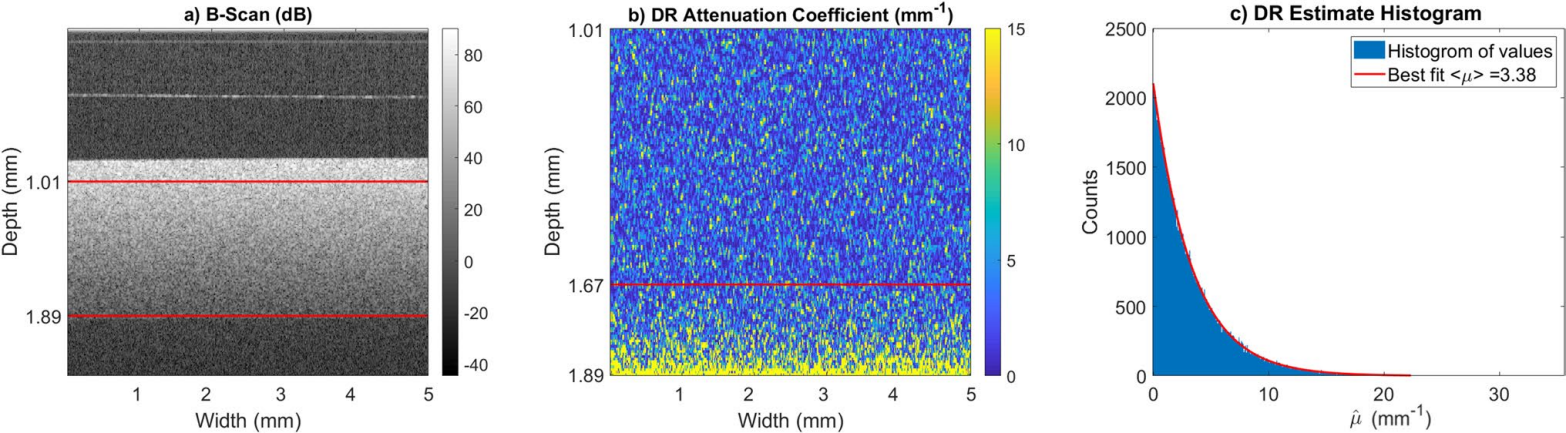
This figure demonstrates the validity of the likelihood function given in Eq. (17) by considering the distribution of attenuation coefficients for a very homogeneous phantom. Both the analysis and figure creation were done in Matlab 2019a^27^, https://www.mathworks.com/. (**a**) An OCT B-scan of a phantom made by suspending silica beads with mean diameter of $0.47 {\text { micron}}$ and a refractive index of 1.425 in water which has a phase refractive index of 1.32^28^. The red lines indicate a homogeneous region where the DR estimate is made. (**b**) The DR estimate for the attenuation coefficient of the B-Scan shown in (**a**). The overestimation artifact is clear towards the bottom part of the scan. (**c**) Histogram of estimated values for the top 100 rows of pixels of the DR estimate on the B-scan. Because the phantom is very homogeneous we expect the histogram to follow Eq. (17) for this region. The exponential fit is in good agreement with the theoretical predicted value of $3.2 {\text { mm}}^{-1}$.

To verify the likelihood model from Eq. (17), the DR attenuation formula is applied to phantom data and a histogram is computed to compare against theory. The data was collected with a Santec IVS 2000 swept source OCT system with a central wavelength of 1309 nm, axial resolution of $12 {\text { micron}}$ in air and lateral resolution of $25.5 {\text { micron}}$. The phantom was made by suspending silica beads manufactured by BaseClear with mean diameter of $0.47 {\text { micron}}$ and a refractive index of 1.425 in water at a volume fraction of 0.08. Water is assumed to have a phase refractive index of 1.32 and a group refractive index of 1.34^28^. Using Mie theory, the scattering cross section is given by $1.9\times10^{-9} {\text { mm}}^2$ and the total attenuation coefficient is $3.2 {\text { mm}}^{-1}$^20^. This value is realistic for tissue^21,29^. An OCT B-scan of the phantom is shown in Fig. 2a. Using these values and Eq. (22) we can see that the expected variance for the attenuation coefficient is $\langle \mu _{oct} \rangle \cdot \zeta = 0.0020 {\text { mm}}^{-2}$ which is very small when compared with the variance of the exponential distribution which is $\langle \mu _{oct} \rangle ^2 = 11.5 {\text { mm}}^{-2}$. Since the speckle variance dominates the distribution of attenuation coefficients the reconstruction should look like Eq. (17). This is demonstrated in Fig. 2c.

**Figure 3:**
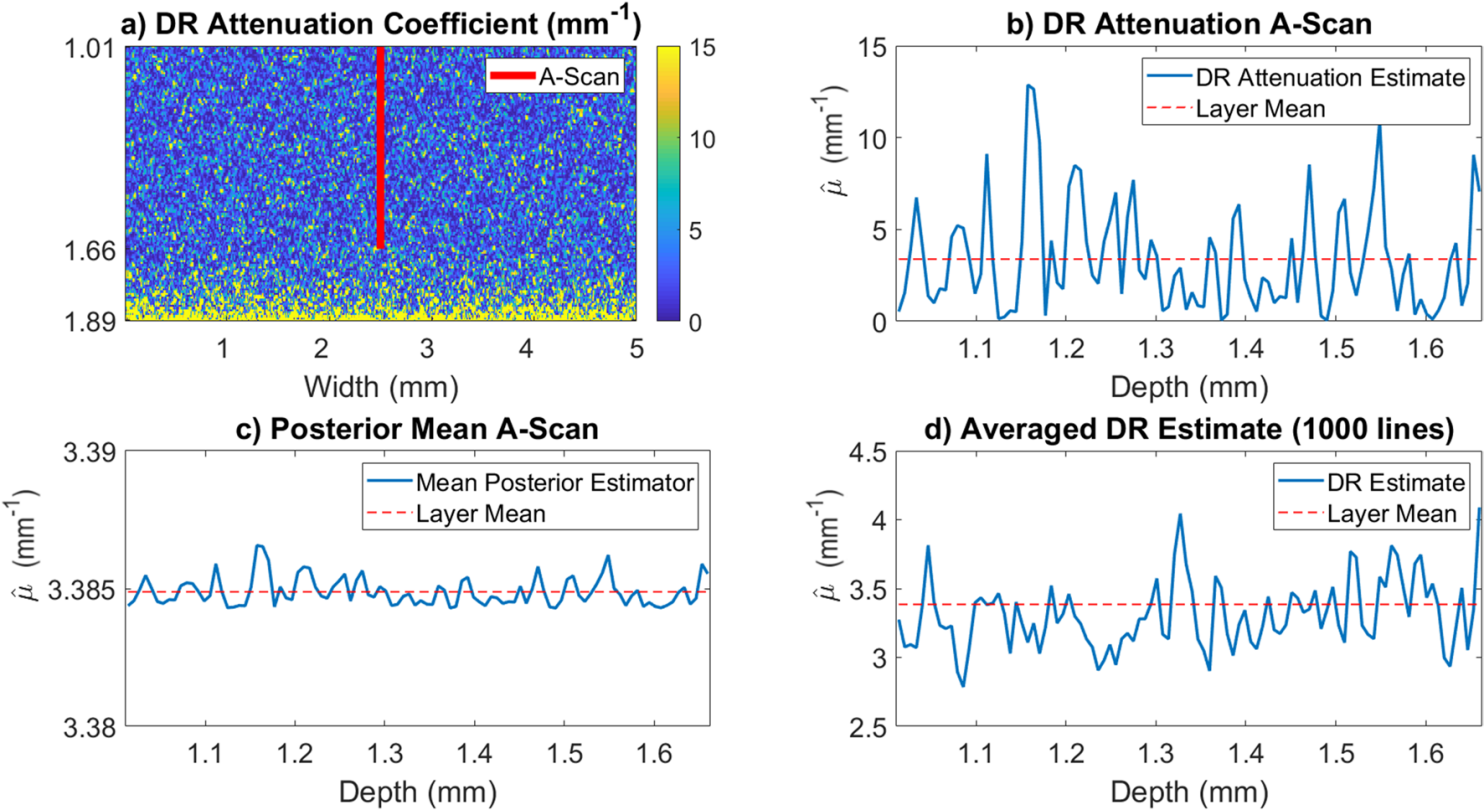
This figure demonstrates variability of the DR attenuation estimates in the presence of speckle and the Bayesian estimator for the attenuation coefficient given in Eq. (26). Both the analysis and figure creation were done in Matlab 2019a^27^, https://www.mathworks.com/. (**a**) The DR estimate for the attenuation coefficient of the B-Scan is shown in Fig. 2. The red line is the portion of the scan considered in (**b**). (**b**) Plot of the DR estimate for the A-scan extracted from (**a**). This estimate shows how highly variable the DR attenuation coefficient appears to be in the presence of speckle. (**c**) Mean of the Posterior estimate for the extracted A-scan. This was computed using the $\langle \mu _{oct} \rangle =3.38 {\text { mm}}^{-1}$ which is the layer mean for the first 100 rows of pixels of the B-scan. The value of $\zeta$ was computed to be $\zeta =6.0053\times10^{-2} {\text{ mm }}^{-1}$ from a voxel volume of $3.2\times10^{-6}$ micron and a scattering cross section of $\sigma _{scat}=1.9\times10^{-9} {\text { mm}}^{2}$. (**d**) Comparative DR estimate for the average of all 1000 A-lines in the B-scan. The resulting fluctuations are still very large even after averaging 1000 A-scans.

Figure 3 demonstrates the effect of the posterior mean estimator defined in Eq. (26) when compared with lateral averaging. Fig. 3a,b show the OCT attenuation coefficient B and A-scans respectively generated from the same OCT B-scan used in Fig. 2. This phantom is very homogeneous so we expect that the variation is almost entirely generated from speckle, thus it is reasonable to assume if sufficiently many A-scans are averaged together then the resulting attenuation coefficient should look constant. Figure 3d shows the resulting OCT attenuation coefficient after laterally averaging 1000 A-scans together. Figure 3c shows the result of the mean estimator defined in Eq. (26) applied to the A-scan from panel (b). There is little remaining variation in the signal when compared with standard lateral averaging.

### Simulation results

**Figure 4:**
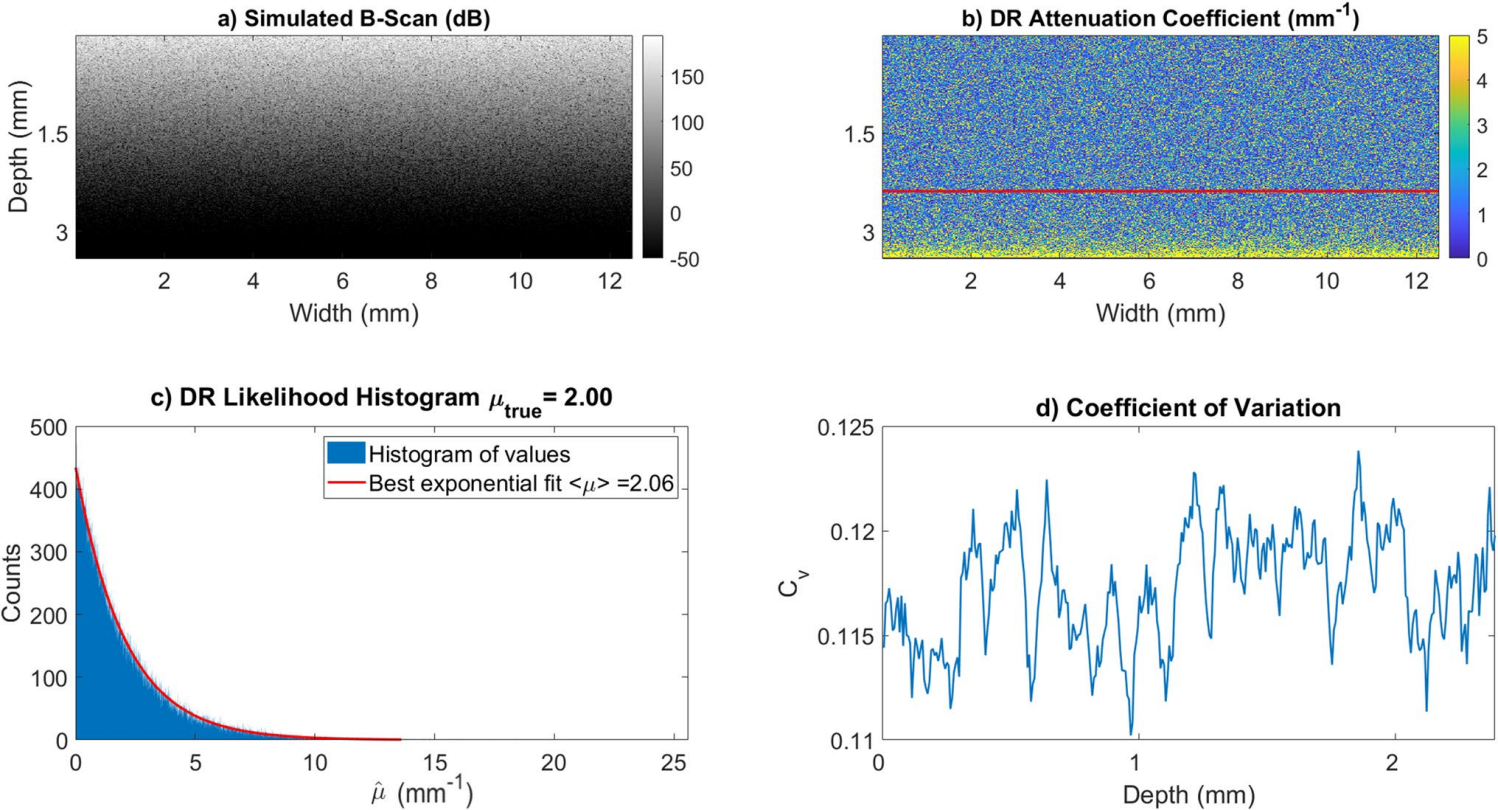
This figure demonstrates the accuracy of the likelihood model and estimates the coefficient of variation of the signal to verify the assumptions made in (14) for simulated signals. Both the simulations and figure creation were done in Matlab 2019a^27^, https://www.mathworks.com/. (**a**) This panel is a simulated B-Scan, which was simulated using parameters $\beta _{NA} = 0.3$, $I_{inc} =1\times10^7$, $\mu _{oct}=2.00 {\text { mm}}^{-1}$, lateral resolution of $\Delta x= 0.022 {\text{ mm }}$ and $\Delta z = 0.0068 {\text { mm}}$ in a domain which is $3.4 {\text { mm}}$ deep. (**b**) This is the DR reconstructed coefficient map. The reconstruction is highly variable around the true attenuation value of $2.00 {\text { mm}}^{-1}$. This panel also demonstrates the growth artifact in the bottom $30\%$ of voxels where estimated values which are much higher than the true value. The estimates below the red line are truncated to avoid the exponential grown artifact. (**c**) This figure is a histogram of the top $70\%$ of pixels from (**b**). As shown in Eq. (17) we expect this to be exponentially distributed with parameter 2.00. A best fit exponential demonstrates this is accurate to three significant figures. (**d**) Coefficient of Variation for the simulated OCT A-scans in panel a) at different depths. In these simulations, $C_v$ stays near 0.12 at all admissible depths.

To validate and better understand the statistical model from “Methods”, a series of simulations were preformed. In Fig. 4a, a B-Scan was simulated using Eq. (2) with $\beta _{NA}=0.3$, $I_{inc}=1\times10^7{ \text{ w/m}}^2$, lateral resolution of $\Delta x= 0.022 {\text{ mm }}$ and $\Delta z = 0.0068 {\text { mm}}$ in a $3.4 {\text { mm}}$ deep domain with a fixed attenuation coefficient of $\mu _{oct}=2.00 {\text { mm}}^{-1}$. Once the deterministic signal is modeled we generate the OCT signal per voxel as a realization of an exponential random variable with parameter given by the true coefficient as in Eq. (6). This random realization can be seen in Fig. 4a. The attenuation coefficient was estimated using the DR method given in Eq. (8) and is shown in Fig. 4b. The reconstruction equation becomes inaccurate near the bottom of the measurement volume, preventing accurate estimation. To avoid these inaccuracies the deepest $30 \%$ of the reconstructed attenuation coefficients were truncated. The $30\%$ value was arrived at by inspection. In Fig. 4c we fit an exponential model to the histogram of the reconstruction and see that the best fit parameter agrees with our model to the 2nd decimal point. In Supplemental Information S2 we show that the truncation error from Eq. (14) leads to an error in the variance of $\hat{\mu }$ on the same order as our fit error.

**Figure 5:**
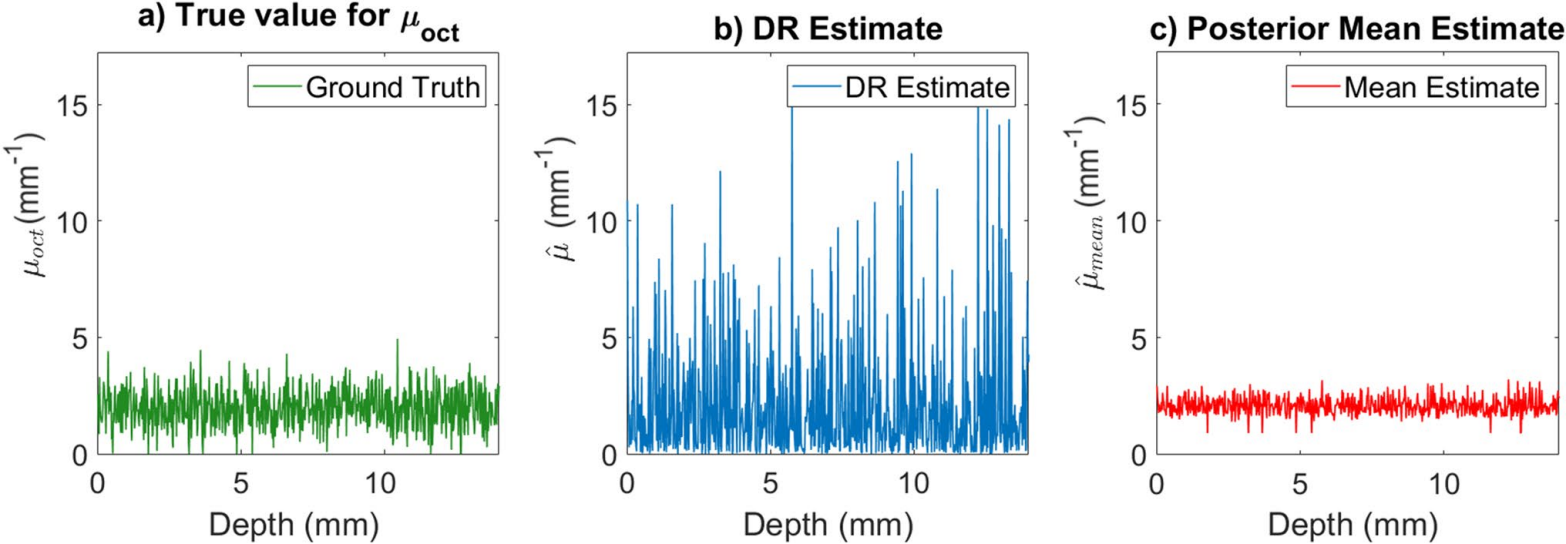
This figure shows estimates of the attenuation coefficient for simulated OCT data using the standard DR and the Bayesian estimator given in (26). The OCT data was simulated with parameters $I_{inc} =1\times10^7$, $\mu _{oct}=2.00$ $\beta _{NA} = 0.3$, $\sigma _{scat}= 1\times10^{-6} {\text { mm}}^2$, a lateral resolution of $\Delta x=0.02 {\text { mm}}$ and $\Delta z = 0.0068 {\text { mm}}$ in a domain which is $13.6 {\text { mm}}$ deep. After the attenuation coefficient was inferred using the DR method the bottom $30\%$ of pixels are discarded to avoid reconstruction artifacts. Both the simulations and figure creation were done in Matlab 2019a^27^, https://www.mathworks.com/. (**a**) This panel shows the ground truth attenuation coefficient for the simulation. This ground truth is a realization of the prior distribution given in Eq. (22). (**b**) This image shows the reconstructed attenuation coefficient using the DR method given in Eq. (8). (**c**) This panel shows an estimate attenuation coefficient given by the mean of the posterior distribution. This estimate was computed using Eq. (26).

To avoid artifacts the bottom $30\%$ of the predicted attenuation coefficient is discarded. Figure 5c shows a posterior mean estimate for the attenuation coefficient which was computed with Eq. (26) voxelwise. In general, the mean attenuation coefficient for the layer, $\langle \mu _{oct} \rangle$, would not be known ahead of time to compute the prior distribution. To account for this, we used the mean of the truncated DR attenuation estimate for the whole scan in Eq. (26). The estimate given by the mean of the posterior distribution for the attenuation coefficient can give much more accurate estimates for the true coefficient than using the standard DR technique, as demonstrated in Fig. 5.

**Figure 6:**
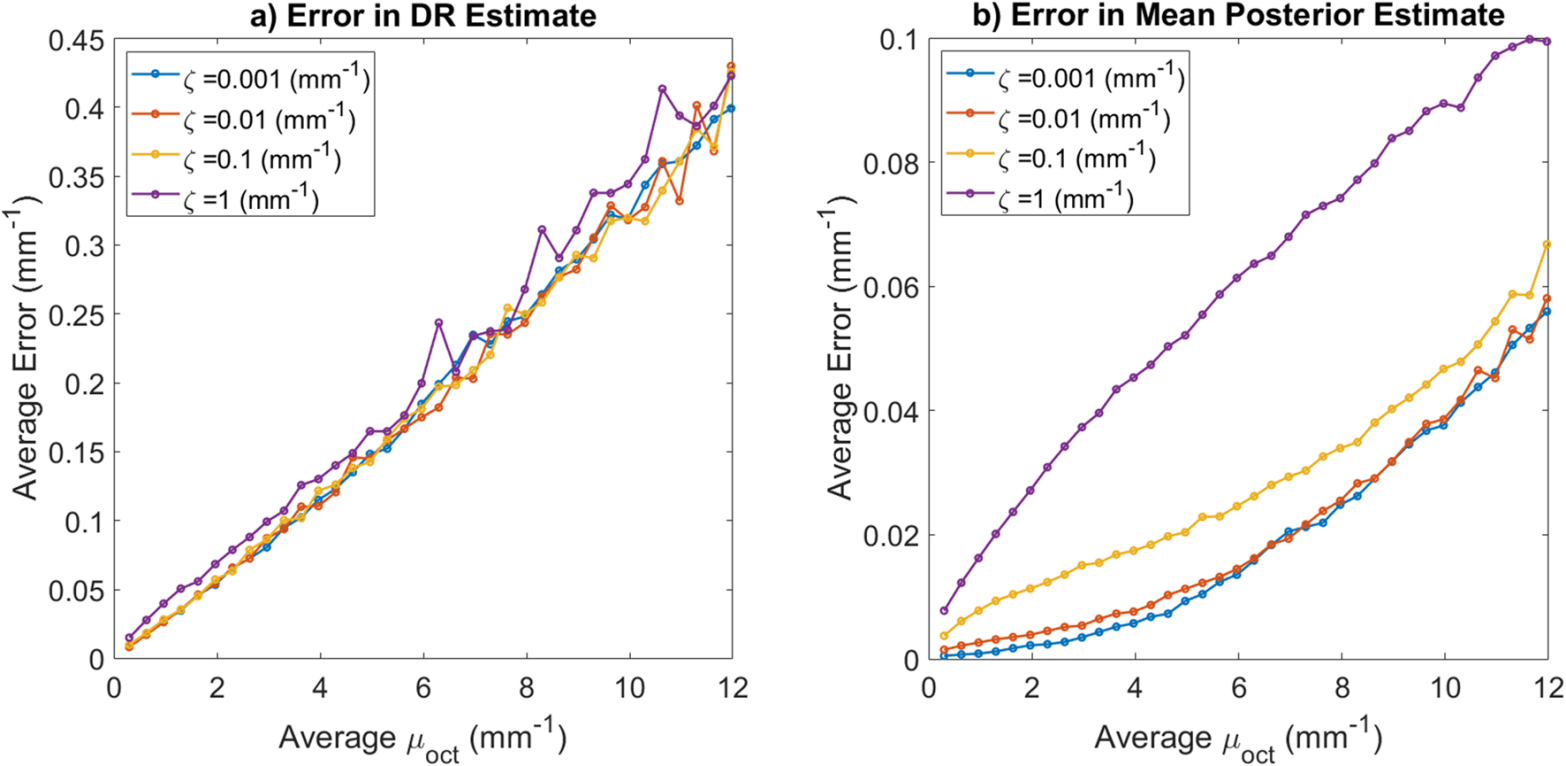
This figure demonstrates how problem parameters impact the error in the attenuation coefficient recovery. For each data point a single A-scan was simulated with 2000 depth measurements. After making the initial DR estimate, the deepest 30% of the pixels were discarded to avoid artifacts. The error (L2) estimates were made for each of the 1400 remaining pixels and averaged. Both the simulations and figure creation were done in Matlab 2019a^27^, https://www.mathworks.com/. (**a**) This image shows how the DR reconstruction error varies with different mean attenuation coefficients for a variety of $\zeta$ values. The $\zeta$ value does not appear to greatly impact DR Reconstruction fidelity. (**b**) This image shows how the mean posterior estimator error varies with different mean attenuation coefficients for a variety of $\zeta$ values. This estimate was computed using Eq. (26). Clearly, the incorporation of of this value into the prior impacts our uncertainty in our Bayesian estimate.

## Discussion

In this paper the impact of speckle fluctuations on the depth resolved recovery of the OCT attenuation coefficient has been addressed. When making an OCT measurement, effectively random voxelwise intensity fluctuations are present in the signal due to speckle, and as a result, the voxelwise mean attenuation coefficient can not be exactly determined. Utilizing a statistical understanding of speckle fluctuations and prior physical knowledge, the posterior distribution for the attenuation coefficient was derived from first principles. This probability distribution better characterizes the voxelwise attenuation coefficient because it allows for the weighing of relative likelihoods and the quantification of uncertainty by measuring the variance of the attenuation posterior distribution.

While the statistical framework derived in this paper is general, the applicability is limited by the assumptions made for the underlying depth resolved reconstruction technique. The DR reconstruction technique, given in Eq. (8), requires that the absorption of light be negligible when compared the total amount of attenuated light. This assumption is restrictive in the materials and wavelengths of light the DR technique can be applied to. However, for the materials and wavelengths used in most common biomedical applications of OCT this assumption is valid. Furthermore, when the probability distribution for the reconstructed coefficient in Eq. (17) was derived, it was assumed that the coefficient of variation of the denominator in Eq. (8) is sufficiently small such that the denominator can be treated as constant. This does seem to be valid in numerical simulations and experiments, however, it is not clear if this is generally true.

Additional physical assumptions are made during the derivation of the prior distribution for the attenuation coefficient given in “Constructing a prior distribution”. The prior distribution allows for the use of physical knowledge about the attenuation coefficient to introduce bounds and bias the probabilities towards realistic values. The derivation given in “Constructing a prior distribution” was made assuming the measured object contained uniform idealized scattering particles with no absorption. While this assumption may not hold for most tissue systems, a normally distributed prior is still a safe choice due to the fact that superpositions of random fluctuations tend to look normally distributed. In real tissue, the parameter $\zeta$ in Eq. (24) is difficult to define, as the meaning of the effective scattering cross section is ambiguous. However, it is still reasonable to assume that the true attenuation coefficient is normally distributed around the mean. The variance of the prior must be provided or inferred by other methods. There are techniques to estimate this parameter from the data such as empirical Bayesian methods^30^, however, the implementation of these techniques can be nontrivial and a robust verification must be performed before the method could be used clinically. While this is outside of the scope of this paper, the Bayesian model presented here serves an an initial step towards the goal of estimating these parameters more robustly in tissue, and elucidates the impact of speckle on the recovered coefficients.

The use of physically accurate statistical models for the attenuation coefficient has several potential advantages. The variance of the posterior distribution provides a way to quantify uncertainty in reconstructions. Furthermore, estimation bias from higher order moments of the posterior can be quantified as well. The likelihood ratio statistic^26^ can be computed using the physically accurate likelihood function given in Eq. (17). This statistical test gives a practitioner a sense of how likely a parameter is to fall within a specified range. In situations where a practitioner may want to have a single number to understand the attenuation in a system, the mean of the posterior can be computed as demonstrated in Fig. 5. In Fig. 6 we measure the error in the estimates for both the DR and mean of posterior estimators as the scattering cross section and attenuation coefficient is varied.

Another potential application domain is in OCT image segmentation where attenuation analysis is used to correct for signal decay and as a contrast enhancement tool^13,31^. As we have discussed in this manuscript, the resulting attenuation image can be very highly variable due to the speckle fluctuations in the original signal. If the attenuation image is to be segmented, these fluctuations may lead to segmentation inaccuracies. Denoising algorithms could combine our exponential likelihood with a spatial priors, such as total variation^14^ which would increase the likelihood of the piecewise constant attenuation coefficients. This could be used to improve segmentation accuracy by removing speckle fluctuations from the attenuation image. This approach may be applicable even in the case of absorbing media because image segmentation does not require extraction of accurate attenuation values, only sufficient contrast between layers.

This work is an initial theoretical step towards fully quantifying and characterizing uncertainties in voxelwise OCT attenuation coefficient recovery in order to better understand the resulting estimates. The likelihood function from Eq. (17) accurately models the voxelwise measurement uncertainty of the attenuation coefficient due to speckle. This likelihood function gives insight into the voxelwise statistics of the DR attenuation images. The posterior distribution for the mean value of the attenuation coefficient, given in Eq. (24), allows parameter estimation to be performed in a consistent and reliable manner by using the posterior mean estimator given in Eq. (26). Furthermore, the posterior distribution derived in this paper can be used to quantify the variance in estimates, which gives insight into uncertainty. While this is a promising approach, further research is still needed to find the best way to apply these techniques to clinical practice.

## Supplementary Information

Supplementary Information.
