# Supplementary material for: Bayesian analysis of depth resolved OCT attenuation coefficients
Source: Sci Rep. 2021 Jan 26;11:2263. doi: 10.1038/s41598-021-81713-7 (PMC7838413; doi:10.1038/s41598-021-81713-7)
Supplement: Supplementary file 1 — Supplementary Information. [file 41598_2021_81713_MOESM1_ESM.pdf]

Supplemental Information: Bayesian Analysis of Depth Resolved OCT Attenuation Coefficients

Lionel Fiske[1,2,3], Maurice C. G. Aalders[3,+], Mitra Almasian[3], Ton G. van Leeuwen[3], Aggelos K. Katsaggelos[4], Oliver Cossairt[2,4], and Dirk J. Faber[3,+]

[1]Northwestern University, Engineering Science and Applied Mathematics, Chicago Il., United States
[2]Northwestern University, Computer Science, Computational Photography Lab, Chicago Il., United States
[3]Amsterdam UMC, University of Amsterdam, Department of Biomedical Engineering and Physics, Cancer Center Amsterdam, Amsterdam Cardiovascular Sciences, Amsterdam, The Netherlands
[4]Northwestern University, Electrical and Computer Engineering, Image and Video Processing Lab, Chicago Il., United States
[*]m.c.aalders@amsterdamumc.nl
[+]these authors contributed equally to this work

S1. Upper Bound for the Coefficient of Variation of a Hypoexponential variable

In this supplemental we will verify that the coefficient of variation of a hypoexponential random variable is bounded by 1. Let $\{X_i\}_{i=1}^m$ be independent and exponentially distributed with positive rates λ_i. Then the variable $D = \sum_{i=1}^m X_i$ is hypoexponentially distributed random variable and has an expected value given by $\langle D \rangle = \sum_{i=1}^m \lambda_i$. Similarly, variance is given by

$$var(D) = \sum_{i=1}^m \lambda_i^2$$

by the Bienaymé formula. Since each constituent λ_i is positive, the binomial theorem implies that

$$var(D) = \sum_{i=1}^m \lambda_i^2 \leq \langle D \rangle^2 = \left(\sum_{i=1}^m \lambda_i \right)^2 .$$

Dividing through by the $\langle D \rangle^2$ and taking a square root gives

$$C_v := \frac{\sqrt{(var(D))}}{\langle D \rangle} < 1.$$

S2. First order error in variance

In section 2.3 a first order Taylor approximation is used to find the distribution of reconstructed attenuation coefficient in the presence of speckle. In this section, to get a sense for the error in this approximation the next order distribution is considered. We define,

$$\hat{\mu}^1(N) := \frac{I_n}{\langle D \rangle} \left(1 - \frac{\eta}{\langle D \rangle} \right). \tag{1}$$

where D is defined as in appendix **S1** and $\eta = D - \langle D \rangle$. Finding a closed form for the distribution of the product of two random variables is non-trivial. However, due to the assumption of independence, the first two moments can be computed as the product of the moments of the independent variables. We have that $\langle \frac{\eta}{\langle D \rangle} \rangle = 0$ and $var\left(\frac{\eta}{\langle D \rangle}\right) = \frac{var(D)}{\langle D \rangle^2} = C_v^2$ from the definitions in section 2.3 and appendix **S1**. Using these moments and the independence of I_n and D yields

$$\langle \hat{\mu}^1(N) \rangle = \mu_{oct} \tag{2}$$

$$var\left(\hat{\mu}^1(N)\right) = \mu_{oct}^2(N) - \mu_{oct}^2(N) C_v^2. \tag{3}$$

The result of keeping the next order term is a reduction in measured variance on the order of the square of the coefficient of variation denoted C_v. In the cases where the coefficient of variation is small this reduction is negligible.

In figure 4 the error in the parameter estimate is in the third decimal place which corresponds with the square of the measured coefficient of variation given by $C_v^2 = .014$. Further analysis is needed to prove mathematically that the error will scale according to the square of the coefficient of variation.

S3. Correcting Beam profile/sensitivity roll off

In this section an approach to correcting the OCT signal for system dependent effects is presented. An OCT signal model to account for OCT signal decay, confocal PSF and sensitivity roll off effects was derived by Faber[1,2]. This model defines the OCT signal in depth as

$$I_T(z;\mu_B,\mu_{oct}) = \alpha \cdot T(z-z_f) \cdot H(z) \cdot \mu_B \exp\left(-2\int_0^z \mu_{oct}(\tau)d\tau\right) \tag{4}$$

where $I_T(z;\mu_B,\mu_{oct})$ is the measured signal at depth z, μ_B is the potentially spatially varying back-scattering coefficient, μ_{oct} is the potentially spatially varying attenuation coefficient. The quantity T is the confocal PSF defined by

$$T(z-z_f) = \frac{1}{1+\left(\frac{z-z_f}{2nz_{R0}}\right)^2}$$

where z_f is the focus position in depth and z_{R0} is the apparent Rayleigh length given by

$$z_{R0} = \pi w_0^2/\lambda_0.$$

In this expression, w_0 is the radius of the beam waist, λ_0 is the center wavelength of the OCT system, and n is the refractive index of the media. The sensitivity roll off function is defined by

$$H(z) = \mathrm{sinc}^2(.5\Delta k_{samp})\exp\left(-\frac{\Delta k_{opt}^2 z^2}{8\log 2}\right)$$

is a depth dependent function of wave number where Δk_{samp} is the spectral resolution and Δk_{opt} is determined by the dispersion line width of the spectrometer.

The model used in this paper assumes all of the attenuation comes from scattering events, the frequency of which depend on material properties. However, because a significant amount of signal attenuation comes from the sensitivity roll off and confocal PSF functions applying our model directly to an uncalibrated signal will result in a large overestimation of true material dependent attenuation. Therefore, to use the DR model presented in this paper these functions must be measured and the resulting data must be corrected as

$$I(z;\mu_{B,NA},\mu_{oct}) = \frac{I_T}{H(z)T(z-z_f)}. \tag{5}$$

References

1. Almasian, M., Bosschaart, N., van Leeuwen, T. G. & Faber, D. J. Validation of quantitative attenuation and backscattering coefficient measurements by optical coherence tomography in the concentration-dependent and multiple scattering regime. *J. Biomed. Opt.* **20**, 1 – 11, DOI: 10.1117/1.JBO.20.12.121314 (2015).

2. van Leeuwen, T. G., Faber, D. J. & Aalders, M. C. Measurement of the axial point spread function in scattering media using single-mode fiber-based optical coherence tomography. *IEEE J. Sel. Top. Quantum Electron.* **9**, 227–233, DOI: 10.1109/JSTQE.2003.813299 (2003).
